# Functional data geometric morphometrics with machine learning for craniodental shape classification in shrews

**DOI:** 10.1038/s41598-024-66246-z

**Published:** 2024-07-06

**Authors:** Aneesha Balachandran Pillay, Dharini Pathmanathan, Sophie Dabo-Niang, Arpah Abu, Hasmahzaiti Omar

**Affiliations:** 1 Faculty of Science, Institute of Mathematical Sciences, Universiti Malaya, Kuala Lumpur, Wilayah Persekutuan Kuala Lumpur Malaysia; 2 Laboratoire Paul Painlevé CNRS 8524, INRIA-MODAL, Université de Lille, Villeneuve d’Ascq, France; 3 Faculty of Science, Institute of Biological Sciences, Universiti Malaya, Kuala Lumpur, Wilayah Persekutuan Kuala Lumpur Malaysia

**Keywords:** Functional data analysis, Principal component analysis, Linear discriminant analysis, Landmarks, Shrews, Geometric morphometrics, Computational biology and bioinformatics, Ecology, Mathematics and computing

## Abstract

This work proposes a functional data analysis approach for morphometrics in classifying three shrew species (*S*. *murinus*, *C*. *monticola*, and *C*. *malayana*) from Peninsular Malaysia. Functional data geometric morphometrics (FDGM) for 2D landmark data is introduced and its performance is compared with classical geometric morphometrics (GM). The FDGM approach converts 2D landmark data into continuous curves, which are then represented as linear combinations of basis functions. The landmark data was obtained from 89 crania of shrew specimens based on three craniodental views (dorsal, jaw, and lateral). Principal component analysis and linear discriminant analysis were applied to both GM and FDGM methods to classify the three shrew species. This study also compared four machine learning approaches (naïve Bayes, support vector machine, random forest, and generalised linear model) using predicted PC scores obtained from both methods (a combination of all three craniodental views and individual views). The analyses favoured FDGM and the dorsal view was the best view for distinguishing the three species.

## Introduction

Morphometrics is a fundamental discipline in biological research that focuses on quantitatively describing and analysing the shape and its variations across organisms. Initially centered on basic descriptive measurements, this field has progressed significantly and is currently employing advanced statistical and computational techniques to study shape and size variation^1^. Conceptually, morphometrics can be broadly categorised into two approaches: landmark-based morphometrics which relies on the precise positioning of anatomical landmarks, and outline-based morphometrics which captures the contour of forms through a sequence of pseudo-landmarks^2,3^. As morphometric techniques continue to advance, the selection of appropriate methods becomes crucial for meaningful applications in biological research.

Geometric morphometrics is a popular field for studying morphological variation in biological organisms. It is based on the idea that the shape of an organism can be described by the coordinates of a set of landmarks on its surface. Landmarks are points on the image of the organism that are consistently located in the same place, regardless of the size or orientation of the organism. Generalised Procrustes analysis (GPA) can be applied to raw landmarks to superimpose the landmark configurations using least-squares estimates and rotation parameters^1^. These variables can be used to compare the shapes of different organisms using graphical visualisation of results to track changes in shape over time and to identify the underlying causes of shape variation.

The utility of GM has been demonstrated in numerous studies involving both macro and microfauna. For instance, Moneva et al. highlighted the taxonomic confusion surrounding *Pomacea canaliculata*, a significant rice pest in Asia, by revealing notable sexual differences in shell size and shape^4^. Importantly, the study underscores the utility of GM methods in detecting subtle morphological differences between sexes, thus offering a more nuanced understanding of shape variation in *P. canaliculata*^4^. Similarly, Theska et al. presented a standardised protocol for conducting GM analyses on 2D landmark data sets focusing on stomatal shapes in the model nematodes *Caenorhabditis* and *Pristionchus*, showcasing its adaptability in quantifying shape disparities within and across species^5^. In another study, Phung et al. investigated sexual dimorphism in *Leptopoma perlucidum* land snails using GM, revealing significant differences in shell size between the sexes. These findings underscored the importance of considering sexual dimorphism in taxonomic studies within the *Leptopom*a genus^6^.

The efficiency and versatility of GM are further exemplified in studies such as Maderbacher et al. (2008) which successfully discriminated three populations of the tcichlid fish, *Tropheus moori* using the GM method^7^. Dudzik also used GM to examine the cranial morphology of Asian and Hispanic populations by performing discriminant and canonical variate analyses^8^. The output of the GM analysis revealed significant differences in cranial shapes between the two groups, yet both studies concur that GM serves as a valuable tool for identifying morphological similarities among populations based on cranial morphology. Within morphometrics, craniodental morphology holds particular significance, offering insights into taxonomic discrimination, evolutionary studies, and biomedical implications. Adams and Rohlf highlighted the importance of craniodental morphometrics in elucidating ecological character displacements in *Plethodon* salamanders through landmark-based geometric morphometric analysis (GM)^9^. Slice explored the application of morphometrics in physical anthropology with a focus on craniodental morphonology. This work highlighted the use of landmark-based morphometrics in studying human evolution and practical application in anthropology^10^. These studies not only shed light on the functional adaptations of craniodental structures but also serve as inspiration for further extending the GM technique for the craniodental morphology of this paper. Although the advantages of GM are widely known, an important limitation of the technique is that a sufficient number of landmarks may not be available to capture the geometry of biological organisms. There is a possibility that important shape differences may occur between landmarks^1^.

The study of craniodental morphology in shrews stands out as an invaluable avenue for gaining insights into their evolutionary trajectory, taxonomic classification, and ecological adaptations. Shrews, belonging to the order *Eulipotyphla* are characterised by their small size, insectivorous diet, and rapid metabolism. Despite their small stature, shrews exhibit remarkable diversity in craniodental morphology, reflecting adaptations to different ecological niches and evolutionary pressures. This is evident in the study conducted by Vasil’ev et al., which revealed geographical variability in the shape of the mandible in three shrew species of the genus Sorex using GM. Notably, discriminant analysis of Procrustes coordinates derived using GM reported a high percentage of correct assignment of individual shrews to distinct local taxocenes, further validating the efficiency of this methodology in taxonomic studies^11^. Moreover, Vilchis-Conde et al. reinforced the significance of GM in supporting the taxonomic classification of semifossorial shrews. Their research also revealed that the shapes of the skull, particularly the dentary, are associated with diet specialisation, highlighting the profound impact of morphological variations on functional aspects such as bite force among shrews^12^.

Our study focuses on the craniodental variation among three shrew species: *Crocidura malayana* (Robinson & Kloss, 1911), *Crocidura monticola* (Peters 1870), and *Suncus murinus* (Linnaeus, 1766). Each species occupies distinct ecological niches: *C. malayana*, a medium-sized shrew, thrives in Thailand, Malaysia, and several offshore islands ^13^. This terrestrial species has been documented in both hill and lowland forests ^14,15^. Meanwhile, *C. monticola*, the smallest shrew in the genus *Crocidura* is restricted to forest areas in Malaysia and Indonesia^16^. On the other hand, *S. murinus*, the largest shrew species, is predominantly found in urban areas and the outskirts of forests, with a wide distribution spanning human settlements in the Indian subcontinent and Southeast Asia^17^.

Functional data analysis (FDA) is a statistical methodology used to analyse data that are represented in the form of functions, consisting of entire curves, surfaces, or other continuous functions, rather than discrete sampling points. Functional data analysis is particularly useful when dealing with data that vary continuously over a domain, such as time, space, or wavelength. In the context of our work, the basic idea behind FDA is used to represent discrete sampling points such as landmark coordinates, as a function. This involves creating functional data that encapsulates all the coordinates to represent the entire measured function. Later, models are generated to predict information based on a collection of functional data by applying statistical concepts from multivariate data analysis^18^. Ramsay and Silverman provided a comprehensive introduction to the FDA, covering theoretical foundations and practical applications, including methods for clustering and classification of functional data, which is particularly relevant for grouping similar surfaces or curves in morphometrics^19^. The FDA framework allows better accuracy in parameter estimation in the analysis phase, effective data noise reduction through curve smoothing, and applicability to data with irregular time sampling schedules^18^.

Bookstein introduced landmark methods for analysing shape differences in outlines which can be considered as a precursor to some FDA techniques. Both landmark and outline analysis have been combined in this study to provide a richer description of the overall shape of the human brain using MRI images^20^. Dryden and Mardia primarily focused on statistical shape analysis which also discussed the foundations of landmark shape analysis, including geometrical concepts and statistical techniques that include analysis of curves, surfaces, images, and other types of object data^21^. Functional data analysis considers shapes as continuous functions or curves, allowing for the analysis of shape changes over a continuum such as time or developmental stages.

In our work, the landmark coordinates used in the GM method will be represented as functions. Each sample element is considered as a function under the FDA framework which often defines time, spatial location, or wavelength as the physical continuum. Functional data geometric morphometrics (FDGM) is proposed in this study, which requires steps to perform statistical analysis on signals, curves, or even more complex objects while remaining invariant to certain shape-preserving transformations^22^. The proposed method combines FDA with GM. Unlike multivariate data analysis, FDA accounts for the continuity of curves and models the data within the functional space, rather than treating them as a set of vectors. In this study, we utilise curvature information from shrew skulls to construct a model comprising a set of landmarks serving as endpoints. By employing interpolation techniques across these landmarks, we can create a more refined shape representation. Although our study adopts a discrete point-based format for convenience, these points fundamentally represent a continuous surface. This approach naturally aligns with the FDA perspective, as elucidated by Ramsay and Silverman^19^. To ensure that the functions are well-aligned for geometric features such as peaks and valleys, curve registration^23,24^ or functional alignment^25^ are applied to warp the temporal domain of functions^22^. The FDA framework surpasses its counterparts, including both the landmark-based approach and the set theory approach with principal component analysis (PCA), when applied to a well-known database of bone outlines^26^. The set theory approach is adopted from a methodology outlined in Horgan^27^ which treats shapes as sets^26^. Each position within the image corresponds to a binary variable, indicating whether it belongs to the shape or not. Consequently, the study performed PCA specifically tailored for binary data^26^. Building on Tian's study of FDA in brain imaging analysis^28^, our research aims to explore FDGM's capacity to enhance sensitivity to subtle shape variations through the analysis of continuous function-based shape changes. This is particularly significant for studying species with minor morphological distinctions or monitoring subtle changes in response to environmental factors.

In our study, we transform landmark data into functional data following generalised Procrustes analysis (GPA). Generalised Procrustes analysis employs rigid transformations, including translation, rotation, and scaling, to align landmark configurations, standardising them for comparison^29^. However, this method may not fully address non-rigid deformations or shape changes independent of position, orientation, or size. Consequently, GPA might not capture all aspects of shape variation, particularly those involving local deformations or complex shapes. To address this limitation, we employ FDA to model non-rigid deformations and intricate shape changes undetected by GPA. By analysing shape changes as continuous functions, FDA can identify and quantify subtle variations and local deformations, offering a more comprehensive understanding of shape variation. Moreover, GPA mandates a one-to-one correspondence between landmarks across specimens, simplifying analysis but potentially overlooking true anatomical correspondence, especially when dealing with ambiguous landmarks^29^. In contrast, FDA relaxes this requirement, aligning shapes based on overall shape curves or surfaces rather than exact landmark correspondences. This allows for more flexible matching of shapes, particularly when landmarks are ambiguous or difficult to identify consistently.

We utilise the functional data to perform multivariate functional principal component analysis (MFPCA) to observe variation among three shrew species, comparing the results with principal component analysis (PCA) in GM. Multivariate functional principal component analysis generates principal component scores (MFPC scores), capturing major sources of shape variation among the species. Landmark data sampled from curves are succinctly represented by continuous curves based on the Karhunen-Loève theorem. Our study demonstrates that FDGM can identify shape differences using classification methods, offering insights into underlying factors such as ecology or behavior. While GM standardises landmark configurations effectively, the integration of FDA enhances morphometric analysis by capturing shape variation more comprehensively and sensitively, especially in complex structures like skulls.

We aim to implement the FDGM framework to observe the existence of significant differences in the craniodental shapes of three species of shrews. We organise our study around the null hypothesis that there are no significant differences in craniodental shapes among the three species of shrews under study. Any observed variations are attributable to random fluctuations or measurement errors, rather than indicative of genuine distinctions related to ecological niches or evolutionary processes. The hypothesis is framed within the framework of traditional morphological analyses, which have long been instrumental in understanding evolutionary relationships and ecological adaptations among shrew species. Shrews, being small mammals with diverse habitats and diets, provide an intriguing subject for morphological investigation. Thus, these craniodental differences can be related to the different ecological niches that these three species occupy^30^.

For our analysis, we collected 89 adult shrew specimens: 29 from *S. murinus*, 30 from *C. monticola*, and 30 from *C. malayana*. The habitats of *C. malayana* span diverse locations, including Lata Belatan, Terengganu; Ulu Gombak; Aur Island, Johor; Pangkor Island, Perak; Bukit Rengit, Pahang; Cheras Road, Kuala Lumpur; Port Dickson, Negeri Sembilan; and Dusun Tua, Selangor. Conversely, *C. monticola* exhibits a broader habitat range, inhabiting environments such as Ulu Gombak; Wang Kelian, dominated by secondary lowland forest, and Maxwell Hill, an upper dipterocarp forest, among others. *Suncus murinus*, on the other hand, is observed in locations like Wang Kelian, Perlis; Alor Setar, Kedah; Air Hitam, Pulau Pinang; Lumut, Perak; Ulu Gombak, Selangor; and Bukit Katil, Melaka. These varied habitats likely contribute to the divergence in craniodental morphology between species. Notably, *C. malayana* and *C. monticola* coexist in sympatry in Ulu Gombak, sharing the same habitat or niche. This study aims to elucidate the relationships between these species, offering valuable insights into the evolutionary processes shaping their craniodental morphology.

Morphometric studies for classification and identification tasks are enhanced by extensive machine learning methods^31^. The naive Bayes (NB), support vector machine (SVM), random forest (RF), and generalised linear models (GLM) classification models^32^ are frequently applied because they have been successfully used in many previous studies. In Rodrigues et al., NB was the best classifier for detecting landmarks in automatic wing geometric morphometrics classification of honeybee (*Apis mellifera*) subspecies^33^.Thomas et al. also applied the NB classifier in their study to propose a novel approach in GM to automate morphological phenotyping in ways that capture comprehensive representations of morphological variation with minimal observer bias^34^ which indicates that NB can be a potentially valuable tool for classification and pattern recognition tasks based on shape data. Bellin et al. successfully combined geometric morphometrics with different machine learning algorithms, including SVM with radial basis function (RBF) kernel. This study demonstrated the effectiveness of SVM in correctly classifying two *Anopheles* sibling species of the *Maculipennis* complex based on shape data^35^. Hence, this study aims to incorporate supervised learning, particularly SVM, for the classification of three shrew species based on their morphological features. SVM can be used to classify shapes into different categories based on their landmark coordinates or shape descriptors. In this approach, each classifier separates the points of two different species and combines all one-vs-one classifiers which leads to a multiclass classifier.

Arai et al. applied RF in the context of morphological identification in skulls, specifically between spotted seals and harbour seals, using geometric morphometrics. The study achieved an identification accuracy rate of 100% using RF by narrowing down to a subset of eight key landmarks out of a total of 75 landmarks^36^. The ensemble nature of RF allows it to capture both linear and non-linear relationships in the data, making it robust and accurate for shape classification tasks. The success of RF in morphological identification^35,37,38^ has encouraged this study to compare the effectiveness of this classifier in the classification of the shrew species based on the FDGM framework. Generalised linear models (GLM) serve as extensions of linear models, enabling the accommodation of nonlinearity and non-constant variance within the data. Consequently, GLMs are equipped to handle various data distributions, making them well-suited for analysing species-habitat relationships which often deviate from normal distributions^39^.

## Methodology

### Data description

#### Shrew skull image acquisition

**Figure 1:**
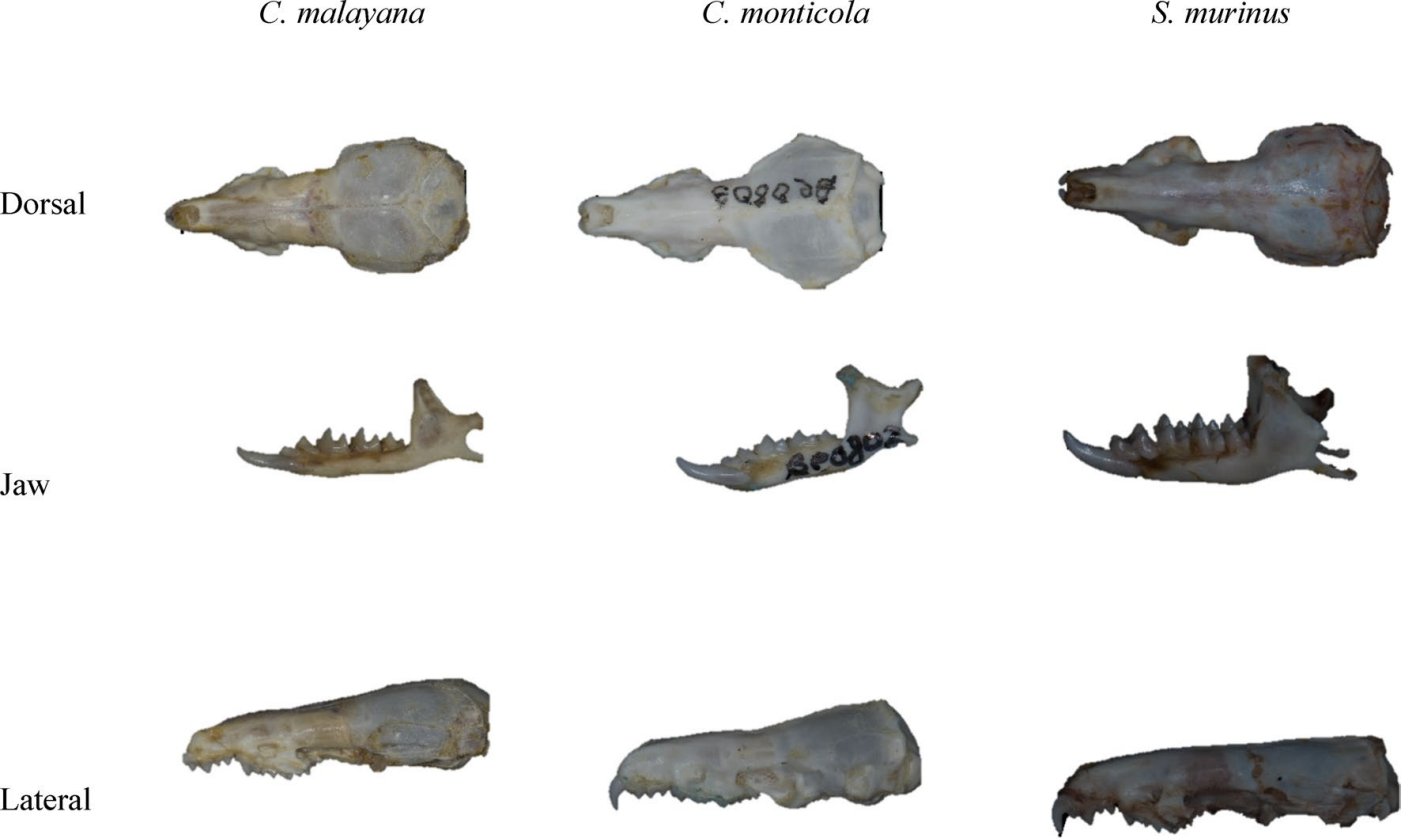
Digital skull images of dorsal, jaw and ventral views of *C. malayana, C. monticola, and S. murinus.*

The skulls of *C. malayana, C. monticola, and S. murinus* can be viewed from different angles, i.e., dorsal, jaws, and lateral depending on the shape of the specimen (Fig. 1). A total of 89 specimens of three different shrew species *(C. malayana, C. monticola, S. murinus)* were retrieved from the Museum of Zoology, Universiti Malaya (UM), Kuala Lumpur, Malaysia. All the skulls extracted from each specimen were separately placed in small bottles for geometric morphometrics. Skull digital images were captured using Nikon D90 with 15× magnification and stored in the Tagged Image File Format (tiff) format with a resolution of 4288 × 2848 pixels. Adobe Photoshop CS6 was also used to improve the image quality^40^.

#### Landmark data acquisition

After the images are acquired, we used TPSUtil32 to obtain the TPS files for all three views. These files were then used in TPSDig2 for landmarking. Replicates were generated by digitising landmarks on each of the shrew images three times. This method ensured consistency and reproducibility by having the same observer capture the landmarks on three separate occasions. By comparing these replicated landmarks, any variation or errors introduced by the observer during the process could be quantified and assessed. The average of these replicated landmarks was subsequently utilised for further analysis.

We analysed the dorsal, jaw, and lateral views of each specimen, with a total number of 25, 50, and 47 landmarks respectively, being sequentially placed on each specimen. These landmarks, consisting of both Type I and Type III collectively form the landmark configuration for each view.

For the dorsal view, 25 landmarks were placed including 16 Type I landmarks (LM1, LM4–LM11, LM13–LM15, LM22–LM25) and 9 Type III landmarks (SLM2–SLM4, SLM12, SLM16–SLM21). Similarly, in the jaw view, 50 landmarks were positioned, comprising 32 Type I landmarks (LM1, LM3-LM22, LM24–LM26, LM32–LM35, LM41–LM43, LM48, LM50) and 18 Type III landmarks (SLM2, SLM23, SLM27–SLM31, SLM36–SLM40, SLM44–SLM47, SLM49).

Lastly, the lateral view consisted of 40 landmarks being Type I (LM1, LM4–LM11, LM15–LM18, LM20, LM22–LM47) and 7 landmarks being Type III (SLM2, SLM3, SLM12–SLM14, SLM19, SLM21).

As suggested by MacLeod (2013), the application of any specific treatment to semi-landmarks, such as the sliding landmark analysis for geometric morphometric analysis has been refrained from this study. This is to prevent any alteration of the original geometric relationships which would complicate the interpretation of the results^41,42^. Our approach aligns with previous craniodental studies on shrews, which similarly avoided specific treatments for semi-landmarks. For instance, White and Searle examined the correlation between genetic diversity and fluctuating asymmetry (FA) in mandibles from island and mainland populations of common shrews on the west coast of Scotland using GM analysis^43^. Similarly, Quintela et al. investigated the geographic variation in skull size and shape among populations of the swamp rat *Scapteromys tumidus* across eight geographic clusters in southern Brazil, utilizing dorsal, ventral, and lateral views of the skull^44^.

The statistical analysis of three views was performed in R version 4.2.1. To use the GM data, the raw coordinates obtained from the landmarks of all three craniodental views were processed using generalised Procrustes analysis (GPA) for optimal registration using translation, rotation, and scaling using the *gpagen* function in the *geomorph* package^45^. Outline methods can produce useful and valid results when suitably constrained by landmarks^46^. This leads to the main idea of this work to incorporate the FDGM approach to observe the separation among the three shrew species.

## Functional data geometric morphometrics

Functional data analysis (FDA) is a methodology employed to examine raw data exhibiting dynamic patterns over time, space, or other intricate dimensions. In this study, the sampling point under consideration comprises landmarks from three craniodental perspectives of shrews, represented as functional forms. The framework has been summarised as follows:
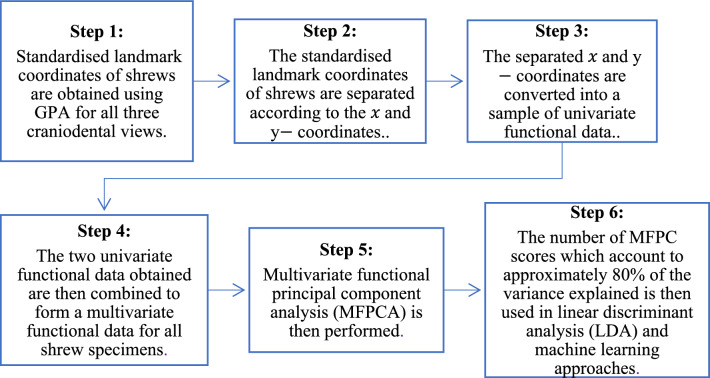


In this study, the sampling point under consideration comprises landmarks from three craniodental perspectives of shrews, represented as functional forms. Each sampling point is vector-valued as two spatial coordinates which are the $x$ and $y-$ coordinates are involved.

**Figure 2:**
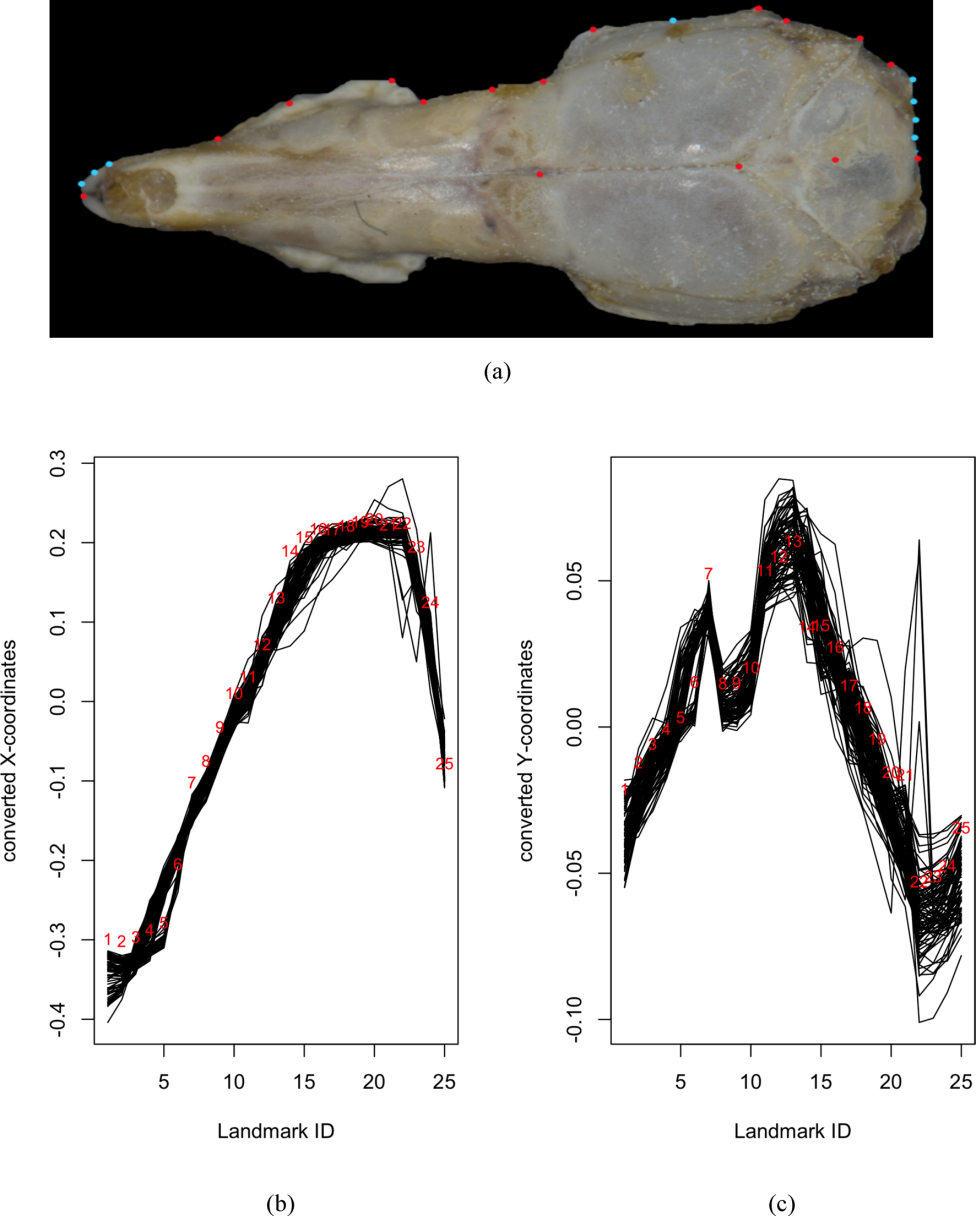
(**a**) 25 landmarks included for dorsal view of *C. malayana*. Landmarks and semilandmarks are represented by red and light blue dots, respectively (**b**) Dimension 1 of converted functional data of the landmark data for the dorsal view using the FDGM method (black lines represent specimens). (**c**) Dimension 2 of converted functional data of the landmark data for the dorsal view using the FDGM method (black lines represent specimens).

**Figure 3:**
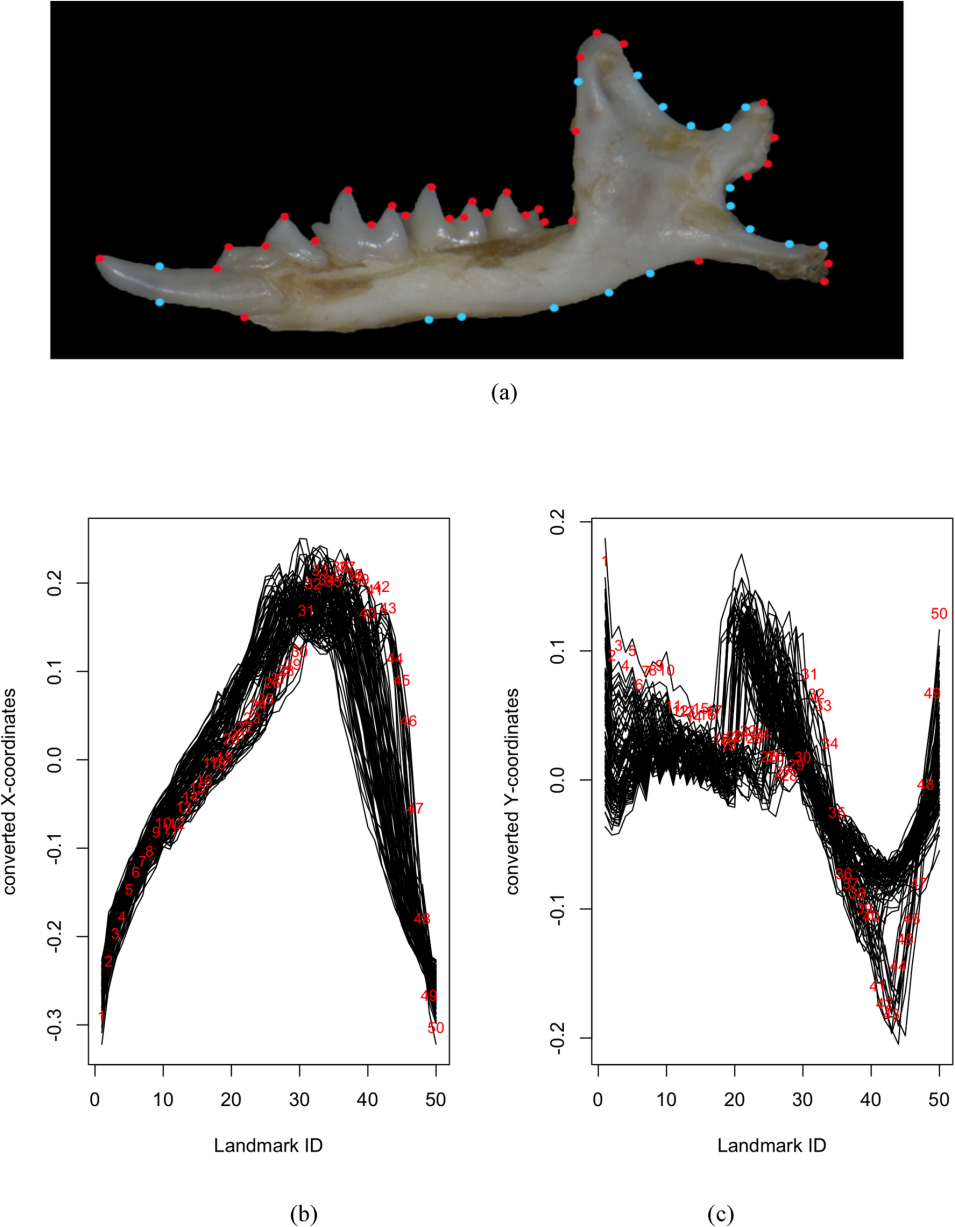
(**a**) 50 landmarks included for jaw view of *C. malayana*. Landmarks and semilandmarks are represented by red and light blue dots, respectively. (**b**) Dimension 1 of converted functional data of the landmark data for the jaw view using the FDGM method (black lines represent specimens) (**c**) Dimension 2 of converted functional data of the landmark data for the jaw view using the FDGM method (black lines represent specimens).

**Figure 4:**
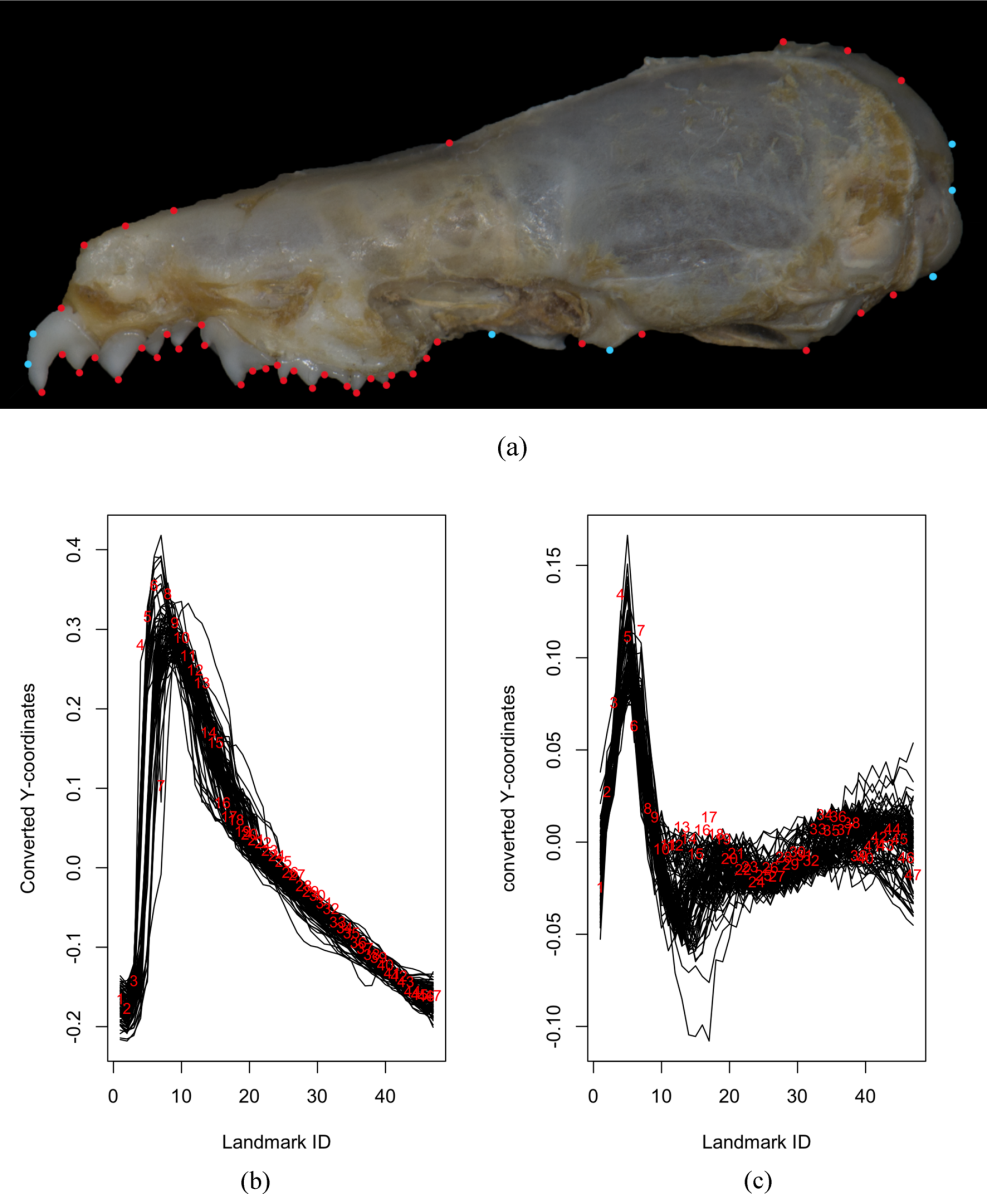
(**a**) 47 landmarks included for the lateral view of *C. malayana*. Landmarks and semilandmarks are represented by red and light blue dots, respectively. (**b**) 2D representation of the $x$ and $y-$ coordinates for the 47 landmarks of crania for the lateral view; (**c**) Dimension 1 of converted functional data of the landmark data for the lateral view using the FDGM method (black lines represent specimens). (**d**) Dimension 2 of converted functional data of the landmark data for the lateral view using the FDGM method (black lines represent specimens).

Let $\left\{{\left( {x}_{k,{\tau }_{1}},{y}_{k,{\tau }_{1}}\right)}^{\text{T}},\dots , {\left( {x}_{k,{\tau }_{N}},{y}_{k,{\tau }_{N}}\right)}^{\text{T}}\right\}$ where $k=1,\dots ,n$ be the standardised landmark coordinates for $n$ specimens and, ${\tau }_{1}, \dots , {\tau }_{N}$ are the observed landmark points with $N$ being the number of landmarks on a dimensional domain (Happ-Kurz, 2020) based on the crania of the shrews (Figs. 2a, 3a, 4a), which will be discussed in detail in Section “Methodology”.

Let ${( {x}_{k,{\tau }_{1}},\dots ,{x}_{k,{\tau }_{N}})}^{\text{T}}$ and ${( {y}_{k,{\tau }_{1}},\dots ,{y}_{k,{\tau }_{N}})}^{\text{T}}$ where $k=1,\dots ,n$, be the separated standardised landmarks for $n$ specimens for the $x$ and $y-$ coordinates, respectively. The raw data is then converted into functions to implement functional data in an object-oriented way. For example, by using the sampling points (boundaries) ${\tau }_{1}, \dots , {\tau }_{N}$ and the set of discrete raw data (values of landmarks) for the $x-$ coordinates, a univariate functional data sample, $\{{X}^{\left(1\right)}\left(\cdot \right), \dots , {X}^{\left(n\right)}\left(\cdot \right)\}$ is constructed (Figs. 2c, 3c, 4c). This is also done for the $y-$ coordinates to construct functional samples, $\{{Y}^{\left(1\right)}\left(\cdot \right), \dots , {Y}^{\left(n\right)}\left(\cdot \right)\}$ (Figs. 2d, 3d, 4d).

The FDGM methodology which is proposed in this work involves multivariate PCA and PCA score-based classification on the landmark coordinates of the craniodental views of shrew specimens.

### Multivariate functional principal component analysis for craniodental views of shrew specimens

The two univariate functional datasets compose multivariate functional data with $n$ outlines, each yielding a vector of $n$ sampling points (landmarks) defined as a $d-$ dimensional functional domain. We used the *MFPCA* package to compute the MFPCA estimates on the multivariate functional data based on their univariate counterparts^47^. Multivariate functional principal component analysis uses the multivariate functional data obtained from landmarks that are independently and identically distributed.

The PCA basis functions are estimated from the multivariate functional data, denoted by $\mathbf{X}$. These basis functions were then applied on $n$ specimens of $\mathbf{X}$ based on the PACE (PCA through the conditional expectation) approach^48^.

Let $\mathbf{X}={\left({X}_{1},{X}_{2}\right)}^{\mathbf{T}}$ be a vector-valued stochastic process that correspond to the functional random variables related to the standardised landmark, $x$ and $y-$ coordinates respectively.

Given the sample of $n$ i.i.d sampling points ${\mathbf{X}}^{(1)},...,{\mathbf{X}}^{(n)}$ of **X**, the estimation procedure for MFPCA involves the following steps:

1. For each element ${X}_{p}$, estimate a univariate FPCA based on the sampling points ${X}_{p}^{(1)},...,{X}_{p}^{(n)}$ by estimating the variance–covariance function ${K}_{p}(\cdot ,\cdot )$. This results in the estimated eigenfunctions ${\widehat{\phi }}_{p,j},$ and scores ${\widehat{\xi }}_{p,j}^{(i)}, i=1,..., n, j=1,..., {J}_{p}$ where ${J}_{p}$ is the number of eigenvalues.
2. Define the matrix **Ξ** $\in {\mathbb{R}}^{n\times J}$ with $J=\sum_{p=1}^{2}{J}_{p}$, where each row $({\psi }_{\text{1,1}}^{(i)},\dots ,{\psi }_{1,{J}_{x}}^{(i)},{\psi }_{\text{2,1}}^{(i)},\dots ,{\psi }_{2,{J}_{y}}^{(i)})$ contains the estimated scores for the $2$ components of the $i$-th sampling point. Consider the matrix $\mathbf{Z}\in {\mathbb{R}}^{J\times J}$ consisting of blocks ${\mathbf{Z}}^{(pq)}\in {\mathbb{R}}^{{J}_{p}\times {J}_{q}}$ with entries
  $${Z}_{jk}^{(pq)}=\text{Cov}\left({\psi }_{p,j},{\psi }_{q,k}\right), j=1,\dots ,{J}_{p}, k=1,\dots ,{J}_{q}, p,q=\text{1,2}.$$

An estimate $\widehat{\mathbf{Z}}\in {\mathbb{R}}^{J\times J}$ of the matrix **Z** is given by

$$\widehat{\mathbf{Z}}=\frac{1}{n-1}{{\varvec{\Xi}}}^{\text{T}}{\varvec{\Xi}}.$$

3. Perform a matrix eigen-analysis for $\widehat{\mathbf{Z}}$ resulting in eigenvalues ${\widehat{\lambda }}_{j}$ and construct the orthonormal eigenvectors ${\widehat{\mathbf{v}}}_{\mathbf{j}}$.
4. Calculate the elements of the estimated multivariate eigenfunctions, ${\widehat{\phi }}_{p,j}\left({t}_{p}\right)$ (${t}_{p}$ is any landmark points for $p$ dimensions) and corresponding multivariate scores, ${\widehat{\xi }}_{j}^{(i)}$.

These estimated eigenvalue functions are derived under the assumption of a finite sample of size $n$ and a finite Karhunen-Loève representation for each univariate function ${X}_{p}$. They are relevant in practice with an appropriate choice of truncation orders.

### Functional linear discriminant analysis for craniodental views of shrew specimens

We applied the multivariate scores, ${\widehat{\xi }}_{j}^{(i)}, i=1,\dots , n;j=1,\dots , K$ where $K$ is the truncated number of components obtained from the landmarks in FLDA to distinguish among the categories studied. The results were compared with the findings observed when PCA is used in linear discriminant analysis (LDA), based on the GM approach. This unsupervised learning approach is a dimensionality reduction technique that is often used to model differences in groups. FLDA provides a possibility to be an efficient tool to improve classification.

The first three $(K=3)$ PC scores were used in LDA for GM and FDGM to compare rates of classification to achieve low classification errors despite a major data reduction^49^. Functional linear discriminant analysis (FLDA) uses a spline curve which is parameterised using a basis function multiplied by a $d-$ dimensional coefficient vector to effectively transform the data into a single $d-$ dimensional space^50^. The classifier also includes the random error to model sampling points from each individual^50^.

The coefficient vector is then modeled using a Gaussian distribution with a common covariance matrix for all classes by analogy with LDA^50^. The observed curves can then be pooled to estimate the covariance and mean for each class which makes it possible to form accurate estimates for each curve based on only a few sampling points^50^.

Let $M$ be the set of classes with $Q$ denoted as the covariance matrix of the variables centered on the class means and $B$ be predictions by the class means^51^. Let $H$ be the $M\times W$ matrix of class means where $W\ge 2$ represents the categorical variables. Denote $G$ to be the $n\times M$ matrix of class indicator variables. Thus, the predictions are $GH$. The sample covariance matrices are as follows.

$W=\frac{{\left(\rho -GH\right)}^{T}(\rho -GH)}{n-M},B=\frac{{\left(GH-1\overline{\rho }\right)}^{T}(GH-\overline{\rho })}{M-1}$, where $\rho$ represents the first three PC scores $\left({\widehat{\xi }}_{j}^{\left(i\right)}, i=1,\dots , n;j=1,\dots , K\right)$ and $\overline{\rho }$ is the mean of $\rho$.

Linear discriminant analysis maximises the ratio of the separation of the class means to the within-class variance by maximizing the ratio $\frac{{a}^{T}Ba}{{a}^{T}Wa}$ where $a$ is the eigenvector of $B,$ corresponding to the largest eigenvalue^52^.

### Classification models

In addition to FLDA, we applied classification methods such as NB, SVM, RF, and GLM to enhance the classification of species among the shrews with the aid of the functional principal component scores (MFPC scores) and PCA scores using the GM method to reduce time complexity. This was done using the *e1071, MASS, and caret* packages in R. The combined analysis of all three views and each separate view was performed. For each view, the data set is split into training and test samples (70:30) and this procedure is iterated 20 times. For each model, the learning is based on the training sample while the performance is assessed by the accuracy based on the test sample. The accuracy and the standard deviation of the 20 iterations are tabulated in Table 1.

**Table 1:** The mean accuracy on the test sample and the corresponding standard deviations (in brackets) based on 20 replications using the MFPCA and PCA scores for (a) dorsal, jaw, and lateral combined; and (b) individual views.

Classifiers	GM	FDGM				
(a) Dorsal, jaw, and lateral combined						
NB	0.848 (0.065)	0.964 (0.031)				
SVM	0.847 (0.071)	0.926 (0.040)				
RF	0.754 (0.057)	0.923 (0.025)				
GLM	0.846 (0.065)	0.964 (0.031)				

Classifiers	GM			FDGM		
	Dorsal	Jaw	Lateral	Dorsal	Jaw	Lateral
(b) Individual views						
NB	0.600 (0.086)	0.481 (0.089)	0.619 (0.061)	0.975 (0.022)	0.563 (0.086)	0.765 (0.065)
SVM	0.541 (0.087)	0.583 (0.054)	0.552 (0.087)	0.959 (0.040)	0.607 (0.096)	0.803 (0.065)
RF	0.535 (0.059)	0.591 (0.093)	0.553 (0.085)	0.957 (0.043)	0.530 (0.076)	0.806 (0.045)
GLM	0.624 (0.089)	0.633 (0.090)	0.609 (0.093)	0.959 (0.036)	0.656 (0.048)	0.657 (0.093)

Brief descriptions of the classification models used on the separate views as well as the combination of all three views are as follows:

(i) Naïve Bayes

The naïve Bayes (NB) classification model is a classifier used to estimate the posterior probability to provide a mechanism that utilises predictors of the training data^53^. Based on the MFPC scores obtained from this study, the Bayes theorem can be written as follows:

$$P\left({c}_{i}|{\widehat{\xi }}_{1}^{\left(i\right)},{\widehat{\xi }}_{2}^{\left(i\right)},{\widehat{\xi }}_{3}^{\left(i\right)}\right)=\frac{P\left({c}_{i}\right)P\left({\widehat{\xi }}_{1}^{\left(i\right)},{\widehat{\xi }}_{2}^{\left(i\right)},{\widehat{\xi }}_{3}^{\left(i\right)}|{c}_{i}\right)}{P\left({\widehat{\xi }}_{1}^{\left(i\right)},{\widehat{\xi }}_{2}^{\left(i\right)},{\widehat{\xi }}_{3}^{\left(i\right)}\right)},$$

where ${\widehat{\xi }}_{1}^{\left(i\right)},{\widehat{\xi }}_{2}^{\left(i\right)},{\widehat{\xi }}_{3}^{\left(i\right)}$ represents the first three MFPC scores and ${c}_{i}$ represents the three shrew species (*C. malayana, C. monticola, and S. murinus*).

(ii) Support Vector Machine

Support Vector Machine (SVM) addresses a multi-class problem as a single “all-together” optimization. This classifier can be used to find a hyperplane in a 2-dimensional space that will separate the scores to their potential species. As this study emphasises 2D, thus the equation of the hyperplane in the two domains can be given as follows:

$$\begin{aligned} y = & \hat{\xi }_{0}^{\left( i \right)} + \hat{\xi }_{1}^{\left( i \right)} x_{1} + \hat{\xi }_{2}^{\left( i \right)} x_{2} \\ = & w_{0} + \mathop \sum \limits_{i = 1}^{2} w_{i} x_{i} \\ = & w_{0} + w^{T} X \\ = & b + w^{T} X \\ \end{aligned}$$

where ${w}_{i}$ = vectors of the first three MFPC scores, $b$ = biased term (${w}_{0}$), $X =$ variables.

The three main hyperparameters in SVM are the cost parameter (C), gamma ($\gamma ),$ and kernel. The cost (C) is the penalty parameter of the error term which controls the trade-off between achieving a low training error and a low testing error. The gamma ($\gamma )$ hyperparameter defines the influence of individual training samples and the kernel is used for mapping the input data into a higher-dimensional space. The radial basis function (RBF) is selected as the kernel function in this study due to its strong classification approach and its versatility in application without requiring prior knowledge of the dataset^54^. SVM-RBF can be defined as follows:

$$k\left({x}_{1},{x}_{2}\right)=\text{exp}(-\gamma {\left|\left|{x}_{1}-{x}_{2}\right|\right|}^{2}),$$

where $$\gamma >0,\gamma =\frac{1}{2{\sigma }^{2}}.$$

(iii) Random Forest

Random forests (RF) is an algorithm for classification developed by Breiman (2001)^36,55^ that is based on bootstrap aggregating or bagging that combines the predictions of multiple decision trees to make a final prediction. This helps to reduce the variance of the individual trees, therefore reducing the overall expected prediction error of the random forest. The working algorithm of the RF classifier is as follows:
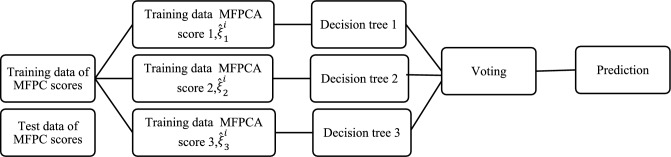

(iv) Generalised Linear Model (GLM): Elastic Net Regularisation

The GLM classifier here is based on the elastic net penalty, which combines both L1 (LASSO) and L2 (ridge) penalties. In the context of geometric morphometrics, elastic net regularisation can be applied to GLMs to control the complexity of the model and prevent overfitting when analysing shape data. In GLM, parameters are assigned to control the L1 and L2 regularisations as well as the strengths of these regularisations. This classifier is based on the MFPC scores which can be represented as ${\eta }_{i}={\beta }_{0}+{\beta }_{1}{\widehat{\xi }}_{1}^{\left(i\right)}+{\dots +\beta }_{i}{\widehat{\xi }}_{3}^{\left(i\right)}$ with a link function that describes how the mean, E $\left({Y}_{i}\right)={\mu }_{i}$ depends on the linear predictor, $g\left({\mu }_{i}\right)={\eta }_{i}$ . The GLM classifier also has a variance function that describes how the variance, $var({Y}_{i})$ depends on the mean, $var\left({Y}_{i}\right)=\phi var\left({\mu }_{i}\right)$ where the dispersion parameter, $\phi$ is a constant.

## Results

**Figure 5:**
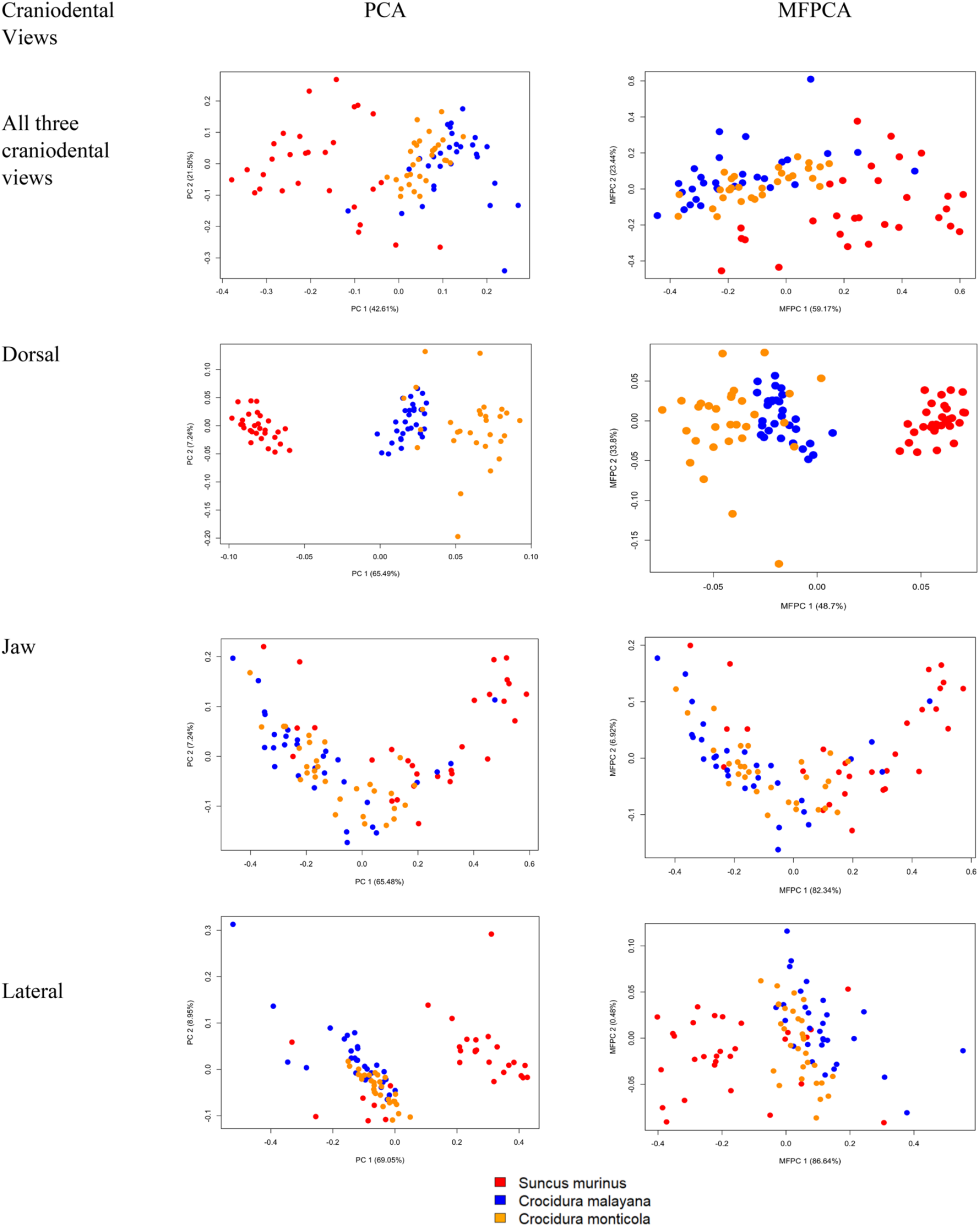
PCA plot using GM method and MFPCA plot using FDGM method for all combined craniodental views, dorsal view, jaw view, and lateral view.

Multivariate functional principal component (MFPCA) using the functional data of all views combined gave a total of 30 eigenvalues. The first two MFPCs accounted for 82.61% of the total variation in the species of shrews. PCA using the GM method yields 88 principal components, where the first two PCs explained 64.12%. The functional principal components show a clearer separation (Fig. 5). Although *S. murinus* does seem to be well separated in the GM method, the approach could not clearly distinguish the other two shrew species.

Thus, employing the FDGM approach has the potential in examining the species variation of the shrews. When PCA is separately conducted on each view, the dorsal view gives the best separation for the three shrew species compared to the other two views for both GM and FDGM methods (Fig. 5).

The dorsal view yielded a total of 9 MFPCs and the first two MFPCs explained 82.44% of the variation among the species. The GM method yields 46 PCs and the first two explained 59.02% of variation. The MFPCA results gave a better separation among the three shrew species compared to the GM method.

There are 10 MFPCs for the jaw view where the first two MFPCs explained 91.41% of the variation in the species. There is a total of 88 classical PCs for the jaw view where the first two explained 77.73% of the variation. As for the lateral view, there is a total of 10 MFPCs and the total variation in species explained by the first two MFPCs is 90.90%. Out of 88 PCs, the first 2 PCs of the GM approach for lateral view explained 78.00% of the total variation. Although *S. murinus* is somewhat separated, the jaw view and lateral view show poor separation for all three species for the GM approach (Fig. 5). A slight improvement in species separation can be observed in the FDGM approach for both views. The performance of the classification models based on individual craniodental views and the combination of all three is evaluated using the first three PC scores of both the FDGM and GM approaches as the first three PCs of all the craniodental views lie within the general rule of thumb threshold of 80% in the FDGM approach. The overall improvement in results for all the classification models when the FDGM approach is applied compared to the GM method is shown in Table 1.

**Figure 6:**
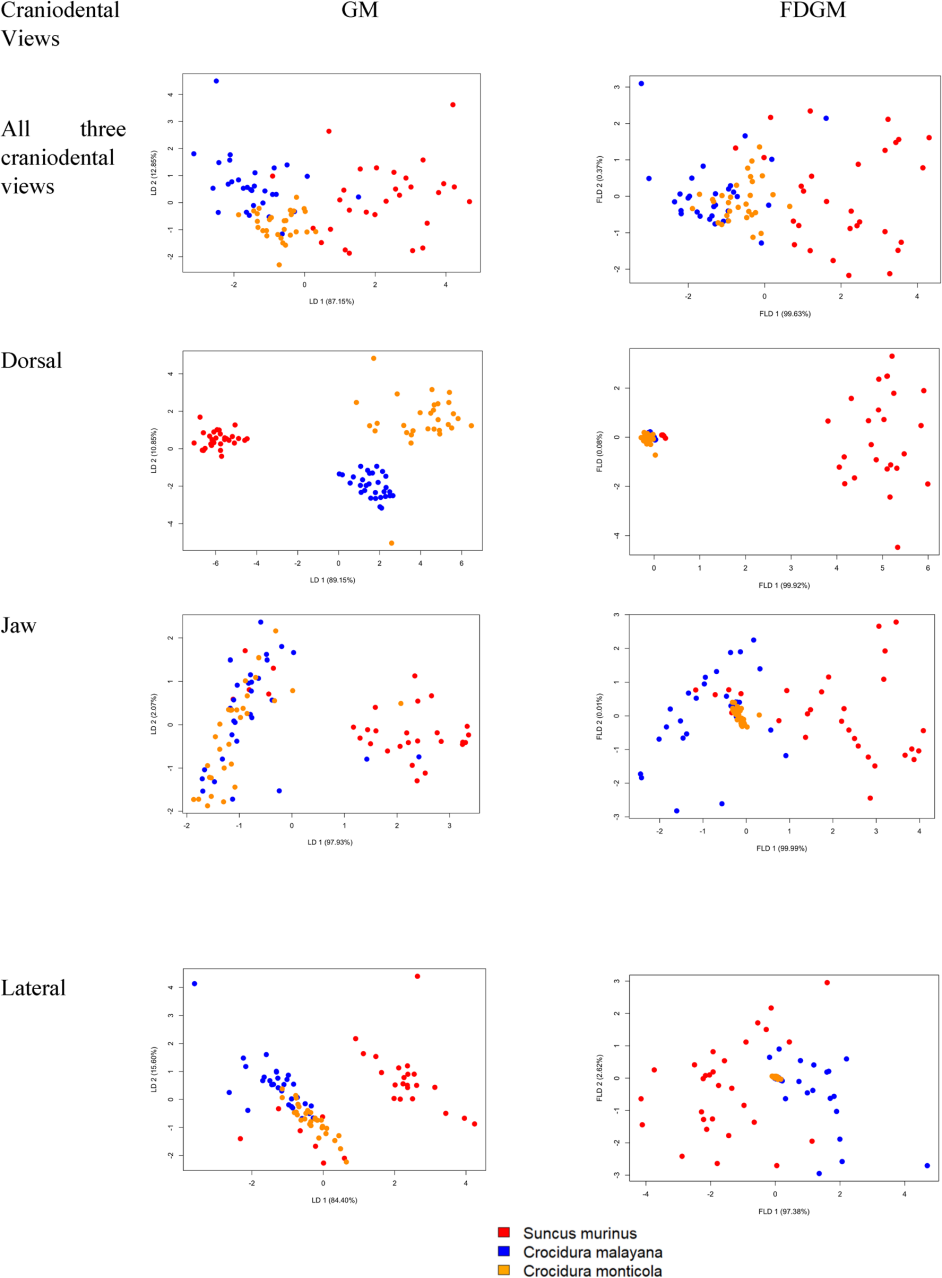
LDA plot using GM method and FLDA plot using FDGM method for all combined craniodental views, dorsal view, jaw view, and lateral view.

The first three PC scores from GM and FDGM were then used in LDA to observe the percentage of separation among the three shrew species based on the craniodental views. Based on GM, the percentage of separations achieved by the first discriminant function is 87.15% and the second discriminant function is 12.85% when all three craniodental views are combined. It is noticeable that the groups are quite well separated with FDGM showing better separation among the three species (Fig. 6) compared to the GM method. The percentage of separations achieved by the first discriminant function in FDGM is comparable to GM, which is 99.9%.

Based on the results obtained in FLDA when the three craniodental views are observed separately, the dorsal view showed a distinct separation of *S. murinus* compared to the other two shrew species, which overlapped (Fig. 6). The clusters among the three species are more compact when FLDA is used compared to the GM method. Based on GM, the percentage of separations achieved by the first discriminant function is 89.15%, 97.93%, and 88.40% for dorsal, jaw, and lateral respectively. The percentages of separation by the first discriminant function showed improvement in the FDGM method, which is 99.92% for the dorsal, 99.9% for the jaw view, and 97.38% for the lateral view. *C. monticola* seems to be well grouped in the FDGM method for all views.

An additional case study, featuring a smaller number of landmarks with a large sample size, is provided in the supplemental materials to showcase the use of the FDGM framework.

## Discussion

We compared the FDGM and GM approaches to study the classification of *S.murinus*, *C. monticola,* and *C. malayana* using craniodental landmarks extracted from the skull images of shrews. Distinct clusters of the shrew species are highlighted when the standardised landmarks of the three craniodental views combined are analysed by FDGM and GM methods. Functional data geometric morphometrics is a better solution as the outlines of the skulls are treated as continuous curves rather than discrete points^56^. High-dimensional data can slow down traditional statistical algorithms which can lead to challenges for standard classification methods when handling such data. Dimension reduction techniques are applied to retain relevant information and reduce correlations, speeding up subsequent analyses while improving accuracy. Functional principal component analysis (FPCA) is commonly used for this purpose and allows for the exploration of data variability in individual curve shapes^18^.

As shown in Fig. 5, PCA based on GM does not give a better separation of the shrew species compared to MFPCA of the FDGM approach; rather, it shows comparable results. When the three craniodental views were individually examined (Fig. 5), the dorsal view showed the clearest separation among the three shrew species using both approaches. This is because the dorsal view gives the most comprehensive view of the skull which includes landmarks from all the major cranial features. Based on the results obtained, this study reveals that the dorsal view of the shrew skulls can be the most informative view for distinguishing between the three shrew species.

The least favorable separations are observed from the jaw view although MFPCA of the FDGM approach shows some improvement and is comparable to the GM method. This may be due to the similarities in *C. monticola* and *C. malayana* as they belong to the same genus. The edges of the molar region tend to be similar for both species. The horseshoe effect present in the GM approach may indicate species turnover along environment gradients^57^. This effect has been commonly observed in ecological ordination obtained by PCA using the GM method^58^. The plots of the MFPCA scores reveal the presence of functional manifolds where the horseshoe effect is noticed^59^. The lateral view also indicates an overlap between the two species and comparable results for both methods. This can be due to the similarity of the back curvature between the two as the region tends to be flat and a little sharp for *S. murinus*. This demonstrates that FDGM can face difficulties in precisely capturing complex and non-linear shape alterations. This is primarily due to the intricate nature of shape transformations in biological structures, which can be influenced by a multitude of factors including genetic variation, developmental processes, and environmental influences.

Considering that the FDGM study relies on functions of craniodental curves based on landmarks, an improvement compared to GM in classification performance for all four models is evident. The dorsal view gives the best rate of classification accuracy among the three views.

Functional data geometric morphometrics integrates principles from FDA with GM methodologies. It treats landmark data as functions, allowing for the analysis of dynamic shape changes over continuous domains. By representing the curves of the three craniodental views as functional forms, FDGM captures the inherent variability and intricacies of morphological changes more comprehensively than discrete landmark-based GM methods. The results obtained from FDGM provide comparable results with the GM methods in analysing dynamic shape changes, which may be challenging for static landmark-based GM methods. In addition, the FDGM framework has the potential to accommodate irregularly sampled or noisy data more effectively, as it does not rely on fixed landmark configurations. However, one potential drawback of this framework would be the requirement of dense and well-sampled data to accurately capture shape dynamics, which may not always be feasible.

## Concluding remarks

In this study, we proposed the use of FDGM on landmark data to represent the shapes of the dorsal, lateral, and jaw of shrew skulls. Results confirm that FDGM improves classification among the three species compared to the GM approach. Particularly, the dorsal view emerges as the best representation for classifying the species in both approaches. The proposed approach utilises data smoothing to represent landmark coordinates as a function derived from raw data, enhancing pattern clarity, and making it a promising tool in morphometrics research. However, FDGM may encounter challenges in accurately capturing complex and non-linear shape transformations. This is because biological structures often exhibit complex shape transformations influenced by a myriad of factors, such as genetic variation, developmental processes, and environmental influences. Capturing these complex shape variations accurately with FDGM may require more sophisticated modeling techniques and larger, more diverse datasets. Additionally, integrating FDA techniques with GM requires careful data preprocessing and analytical methods to mitigate biases or errors. Despite these challenges, FDGM offers a more sophisticated approach to analysing shape variation by modeling shape changes as continuous functions. This departure from traditional discrete landmark-based methods allows for a more comprehensive representation of shape, capturing subtle variations and non-linear transformations more effectively. By exploring the theoretical and practical advancements offered by FDGM, this study aims to contribute to the methodological toolkit of GM and facilitate more accurate and insightful analyses of biological shape data. Additionally, FDGM integrates principles from functional data analysis with geometric morphometrics, providing a more robust framework for analysing shape data. Practically, FDGM enhances the accuracy and sensitivity of shape analysis by enabling the examination of shape changes along continuous curves or surfaces. This can lead to more precise identification of shape differences between groups and a better understanding of shape variation within populations. Future studies can address these challenges and explore the potential of FDGM further. The aim of this study was to assess how FDA can be used to enhance GM. As a result, we avoided specific treatments such as sliding landmark analysis on semi-landmarks in GM. This approach may pave the way for future research to focus on analyses that differentiate between landmarks and semi-landmarks. Additionally, ongoing research on three-dimensional FDGM extensions holds promise for further enhancing morphometrics analysis.

### Supplementary Information

Supplementary Information.

## Data Availability

The images of shrew skulls and TPS files of the three craniodental views that support the findings in this study have been deposited on Figshare (10.6084/m9.figshare.23899332.v2).

## Code availability

The R script allowing to reproduce the entire study is available on GitHub (https://github.com/AneeshaPillay05/2D--Functional-Data-Geometric-Morphometrics.git).
