# Supplementary material for: Functional data geometric morphometrics with machine learning for craniodental shape classification in shrews
Source: Sci Rep. 2024 Jul 6;14:15579. doi: 10.1038/s41598-024-66246-z (PMC11227550; doi:10.1038/s41598-024-66246-z)
Supplement: Supplementary file 1 — Supplementary Information. [file 41598_2024_66246_MOESM1_ESM.pdf]

Supplementary Information

Supplementary Figure S1: Description of positioning landmarks (Type 1) and semi landmarks (Type 2) on shrew images.

Positions on Dorsal View

LM1	Digitised at the anterior most point of suture.
SLM2	Digitised at the anterior most point of suture between nasal and premaxilla.
SLM3	Digitised at the superior most point of nasolachrymal capsule.
LM4	Digitised at the anterior most point of zygomatic plate
LM5	Digitised at the posterior most margin of maximum constriction of antorbital bridge.
LM6	Digitised with equal distance along the point posterior most margin of maximum constriction of antorbital bridge and anterior most margin of the maximum constriction of squamosal root of zygomatic arch.
LM7	Digitised with equal distance along the point posterior most margin of maximum constriction of antorbital bridge and anterior most margin of the maximum constriction of squamosal root of zygomatic arch.
LM8	Digitised at suture between squamosal, alisphenoid and tympanic bulla.
LM9	Digitised at jugal part.
LM10	Digitised along interorbital constriction.
LM11	Digitised along interorbital constriction.
SLM12	Digitised at anterior part of stylomastoid foramen.
LM13	Digitised at lambdoidal ridge.
LM14	Digitised at anterior most point of paraoccipital process.
LM15	Digitised at hypoglossal foramen.
SLM16	Digitised along occipital condyle.
SLM17	Digitised along occipital condyle.
SLM18	Digitised along occipital condyle.
SLM19	Digitised along occipital condyle.
SLM20	Digitised along occipital condyle.
SLM21	Digitised along occipital condyle .
LM22	Digitised at occipital plate.
LM23	Digitised at interparietal.
LM24	Digitised at basioccipital.
LM25	Digitised at palatine.

Positions on Lateral View

LM1	Digitised at tip of incisive tooth.
SLM2	Digitised with equal distance along curvature of incisive tooth.
SLM3	Digitised with equal distance along curvature of incisive tooth.
LM4	Digitised at the edge of incisive tooth.
LM5	Digitised at premaxilla point.
LM6	Digitised along curvature of maxillary.
LM7	Digitised along curvature of maxillary.
LM8	Digitised along curvature of supraorbital foramen.
LM9	Digitised along curvature of occipital plate.
LM10	Digitised along curvature of occipital plate.
LM11	Digitised along curvature of occipital plate.
SLM12	Digitised along curvature of occipital plate.
SLM13	Digitised along curvature of occipital plate.
SLM14	Digitised along curvature of occipital plate.
LM15	Digitised along curvature of occipital condyle.
LM16	Digitised at mastoid point.
LM17	Digitised at auditory bulla point.
LM18	Digitised at pterygoid point.
SLM19	Digitised at hamular process point.
LM20	Digitised at foramen ovale accessories point.
SLM21	Digitised at postpalatine foramen point.
LM22	Digitised along dentary tooth point.
LM23	Digitised along dentary tooth point.
LM24	Digitised along dentary tooth point.
LM25	Digitised along dentary tooth point.
LM26	Digitised along dentary tooth point.
LM27	Digitised along dentary tooth point.
LM28	Digitised along dentary tooth point.
LM29	Digitised along dentary tooth point.
LM30	Digitised along dentary tooth point.
LM31	Digitised along dentary tooth point.
LM32	Digitised along dentary tooth point.
LM33	Digitised along dentary tooth point.
LM34	Digitised along dentary tooth point.
LM35	Digitised along dentary tooth point.
LM36	Digitised along dentary tooth point.
LM37	Digitised along dentary tooth point.
LM38	Digitised along dentary tooth point.
LM39	Digitised along dentary tooth point.
LM40	Digitised along dentary tooth point.
LM41	Digitised along dentary tooth point.
LM42	Digitised along dentary tooth point.
LM43	Digitised along dentary tooth point.
LM44	Digitised along dentary tooth point.
LM45	Digitised along dentary tooth point.
LM46	Digitised along dentary tooth point.
LM47	Digitised along dentary tooth point.

Supplementary Table S2: The variation explained by the first 10 principal components for the MFPCA and PCA scores for: (a) dorsal, jaw and lateral combined; (b) individual views.

Principal Components	PCA	MFPCA
Component 1	0.427	0.870
Component 2	0.203	0.128
Component 3	0.055	0.018
Component 4	0.045	0.005
Component 5	0.030	0.000
Component 6	0.025	0.000
Component 7	0.022	0.000
Component 8	0.018	0.000
Component 9	0.015	0.000
Component 10	0.014	0.000

(a)

Principal Components	PCA			MFPCA		
	Dorsal	Jaw	Lateral	Dorsal	Jaw	Lateral
Component 1	0.391	0.655	0.691	0.484	0.823	0.866
Component 2	0.200	0.072	0.089	0.340	0.069	0.047
Component 3	0.109	0.062	0.050	0.084	0.063	0.045
Component 4	0.063	0.041	0.030	0.044	0.018	0.016
Component 5	0.056	0.033	0.027	0.002	0.011	0.0142
Component 6	0.033	0.021	0.016	0.018	0.007	0.007
Component 7	0.024	0.017	0.013	0.006	0.003	0.003
Component 8	0.018	0.015	0.009	0.002	0.003	0.002
Component 9	0.017	0.010	0.008	0.002	0.002	0.000
Component 10	0.013	0.008	0.007		0.000	0.000

(b)

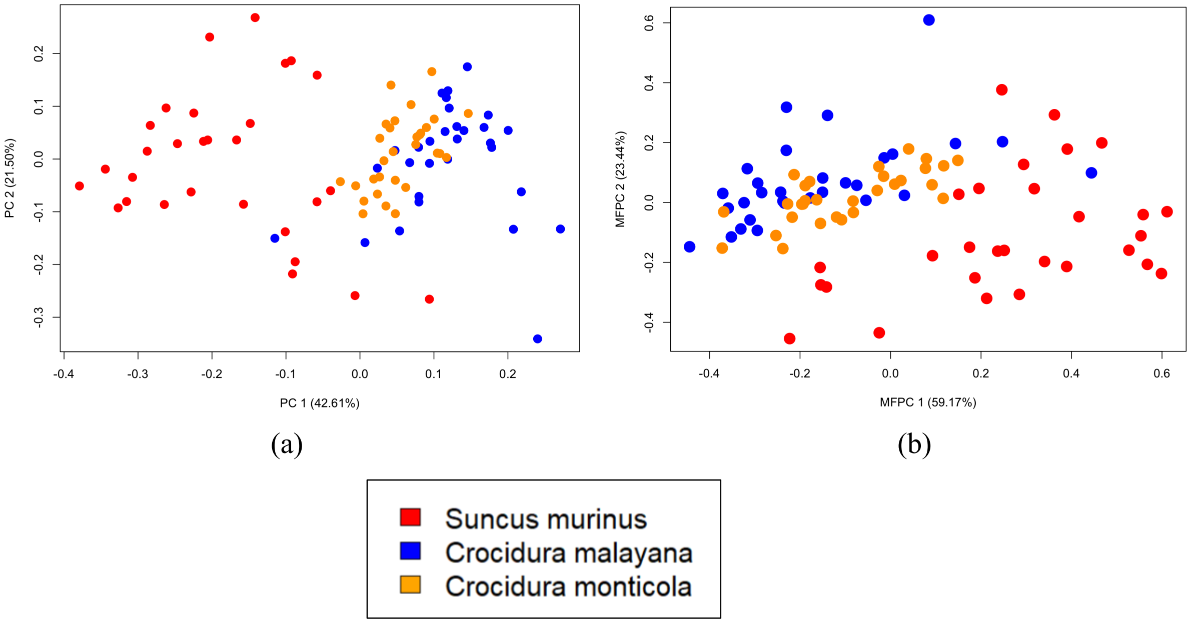

Supplementary Figure S3. The PCs of the (**a**) GM (**b**) FDGM methods for all three views (dorsal, jaw and lateral combined)

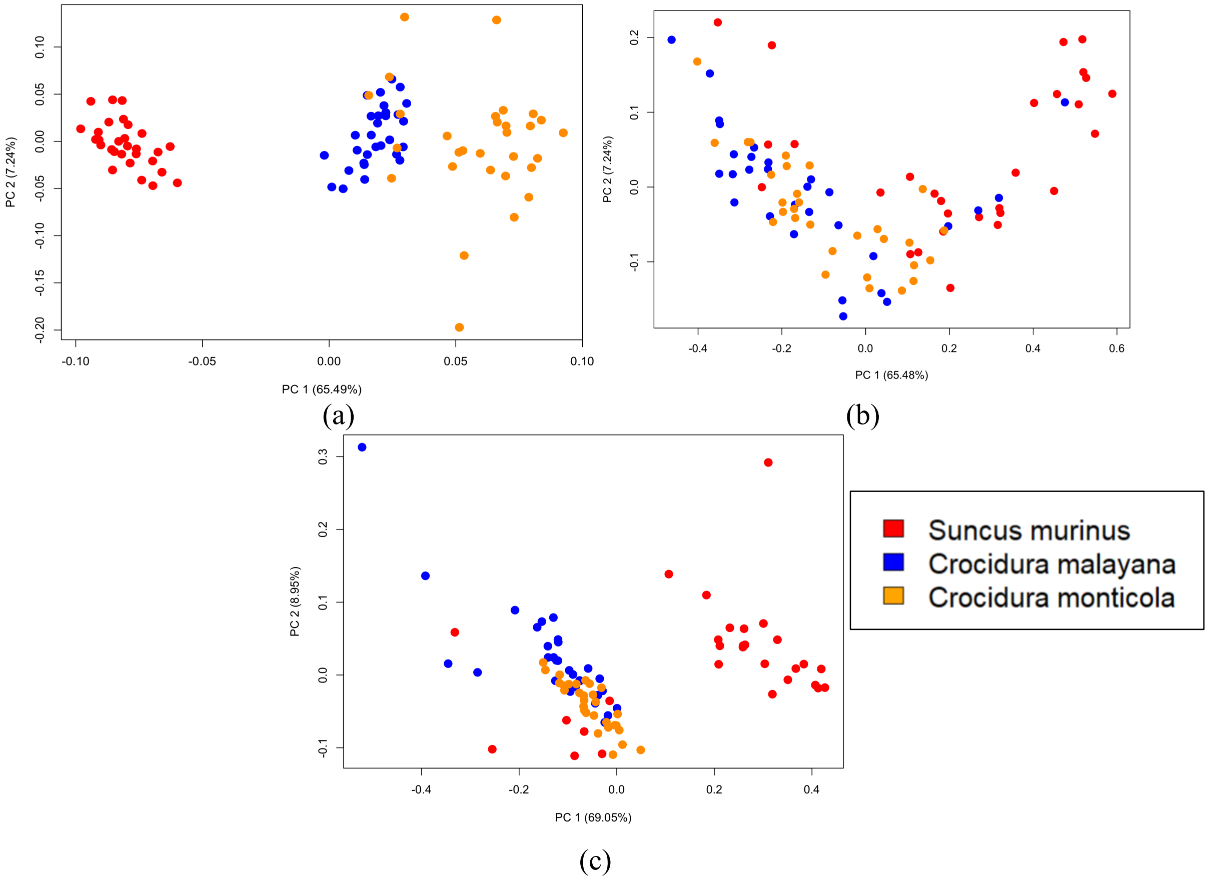

Supplementary Figure S4. PCA plot using GM method for (**a**) dorsal view (**b**) jaw view (**c**) lateral view

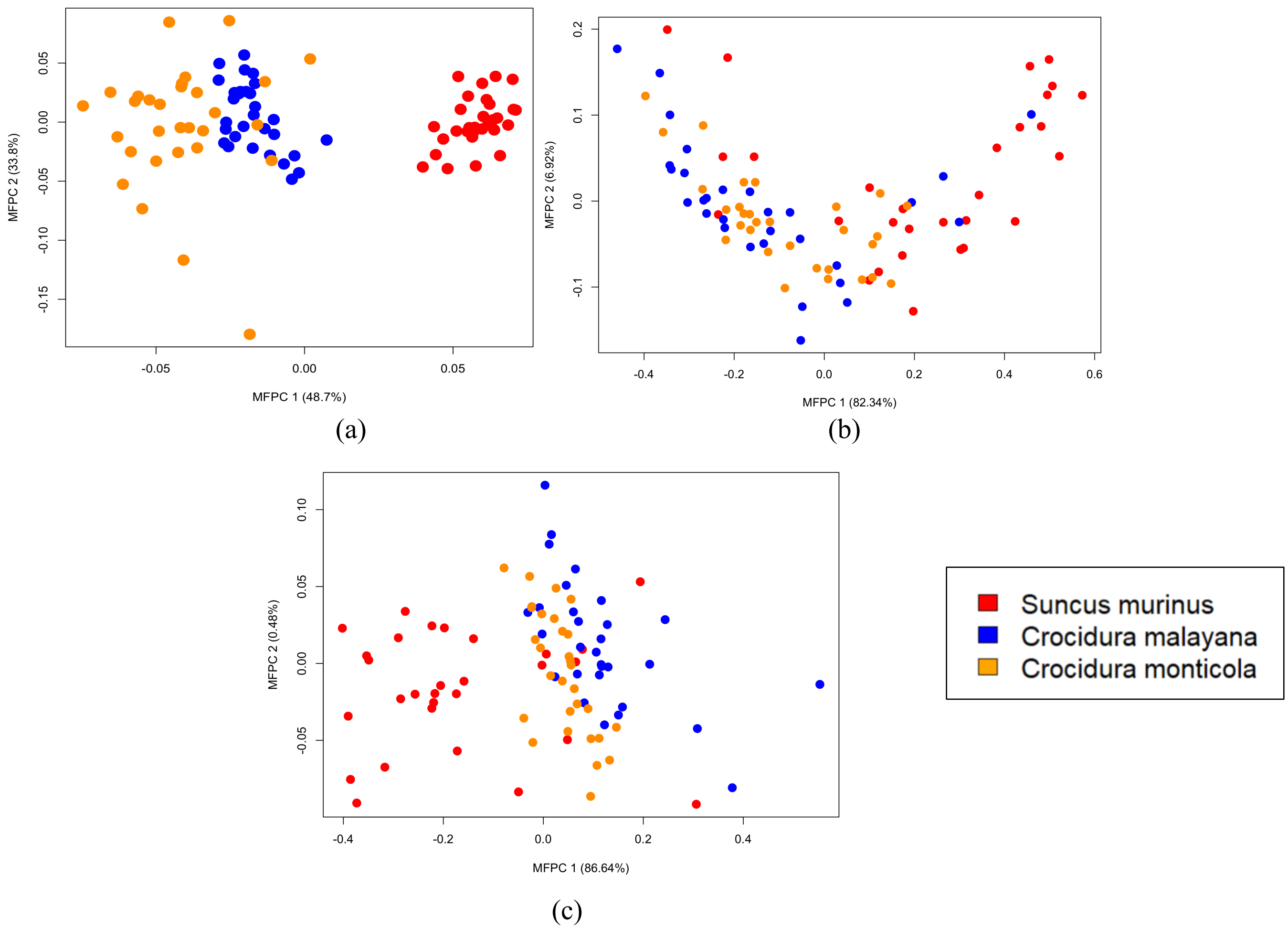

Supplementary Figure S5. MFPCA plot using FDGM method for (**a**) dorsal view (**b**) jaw view (**c**) lateral view

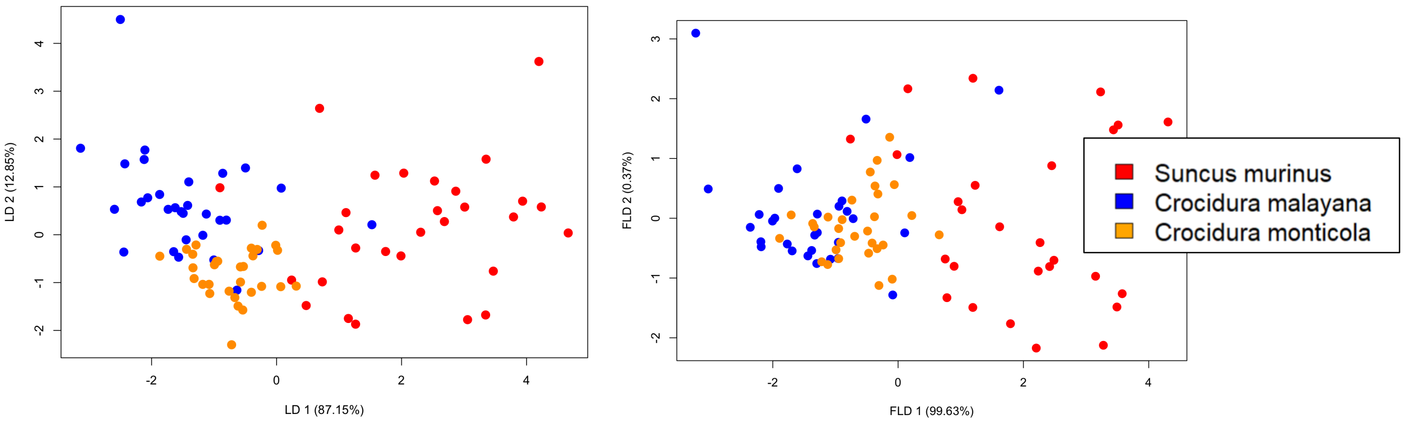

(a) (b)

Supplementary Figure S6. The LDs of the (**a**) GM (**b**) FDGM methods for all three views (dorsal, jaw and lateral combined)

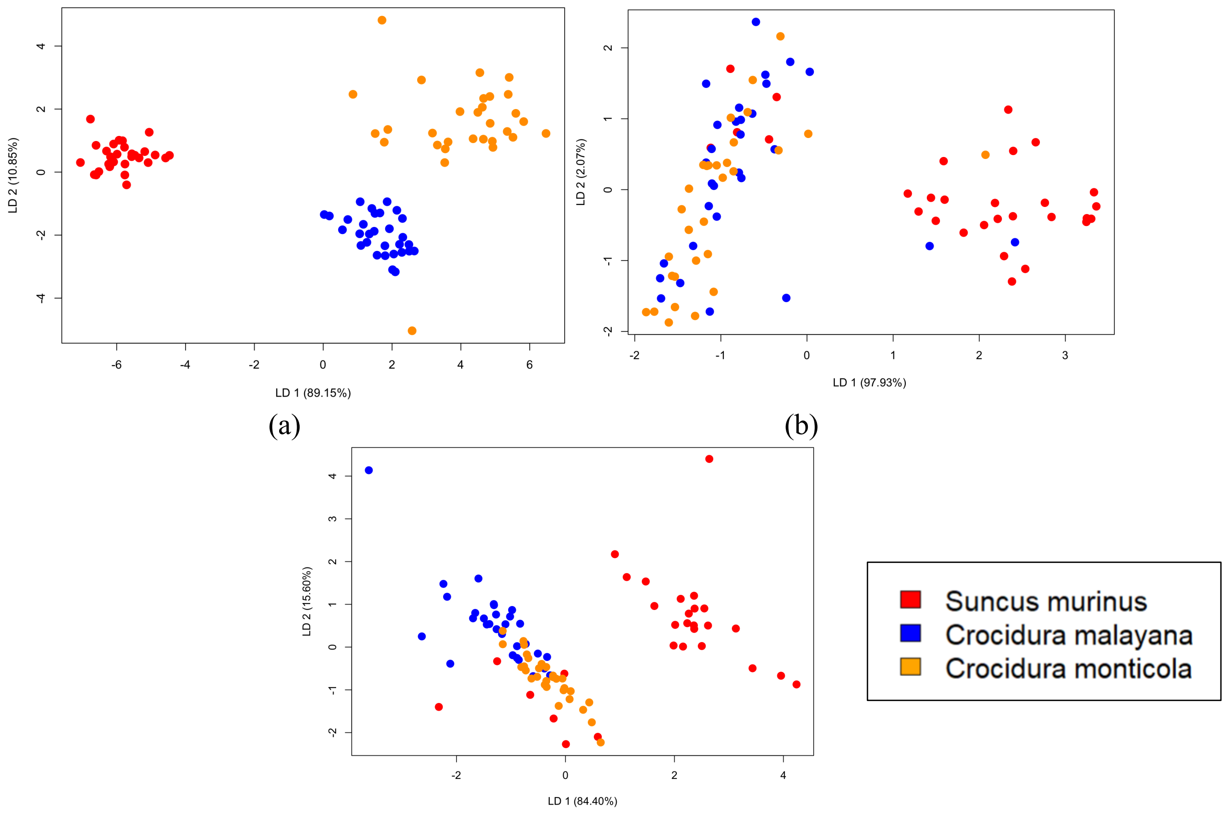

(c)

Supplementary Figure S7. LDA plot using GM method for (**a**) dorsal view (**b**) jaw view (**c**) lateral vi

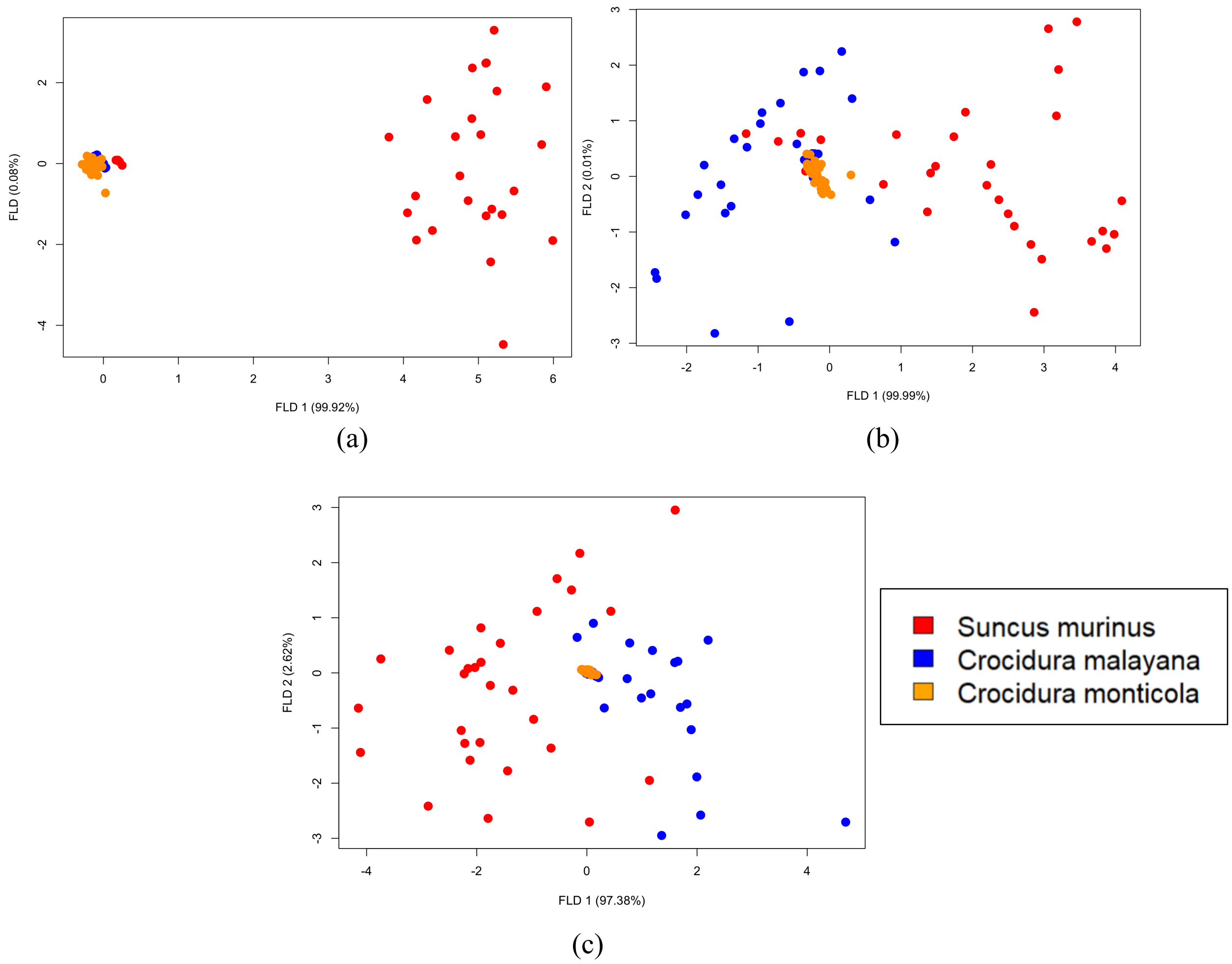

Supplementary Figure S8. FLDA plot using FDGM method for (**a**) dorsal view (**b**) jaw view (**c**) lateral view

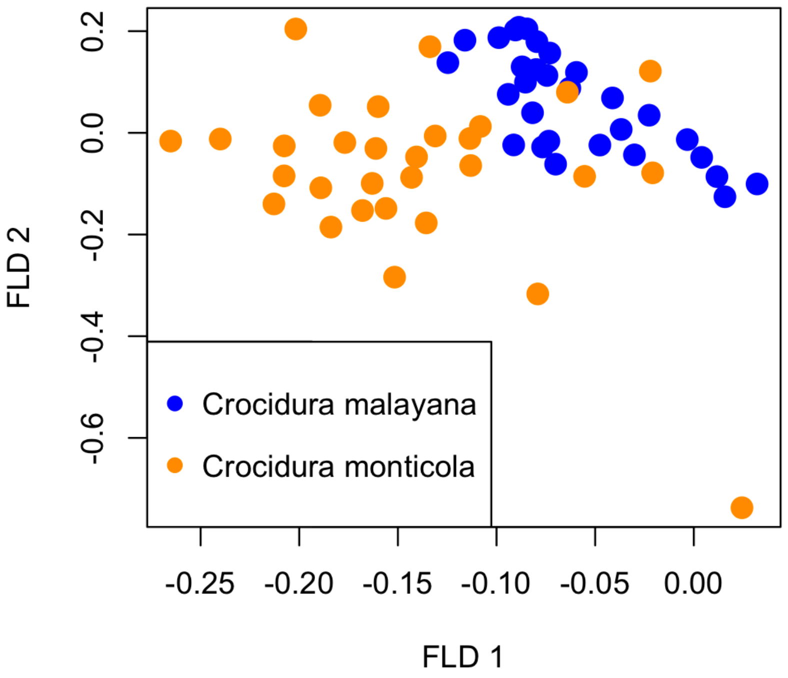

Supplementary Figure S9. Zoomed-in FLDA plot using FDGM method of *C. malayana* and *C. monticola* for dorsal view

Case Study

Data Description

This analysis is based on the data from Khang et al. (2016), utilising anchor morphometry, body size, and morphology data of 13 *Ligophorus* (Monogenea: Ancyrocephalidae) species infecting two marine mugilid (Teleostei: Mugilidae) fish hosts: *Moolgarda buchanani* (Bleeker) and Liza subviridis (Valenciennes) from Malaysia. This case study aims to demonstrate that the methodological framework FDGM can be applied to various types of data beyond craniodental data. The results obtained from the GM and FDGM methods are as follows:

Ventral

PCA **MFPCA**

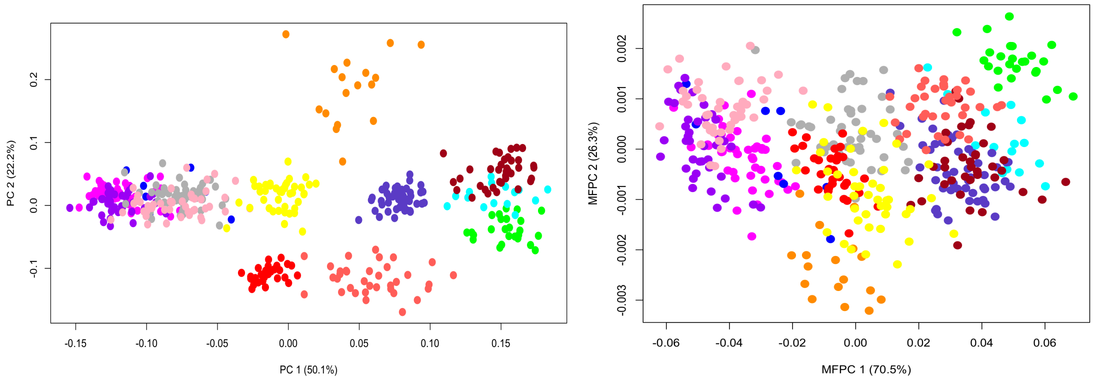

(a) (b)

Supplementary Figure S9. The PCs of the (a) GM (b) FDGM methods.

Results:

- Clusters are better separated using the GM method.
- Variation explained in the first two principal components is greater using the FDGM compared to the GM method.

Ventral (LDA)

LDA **FLDA**

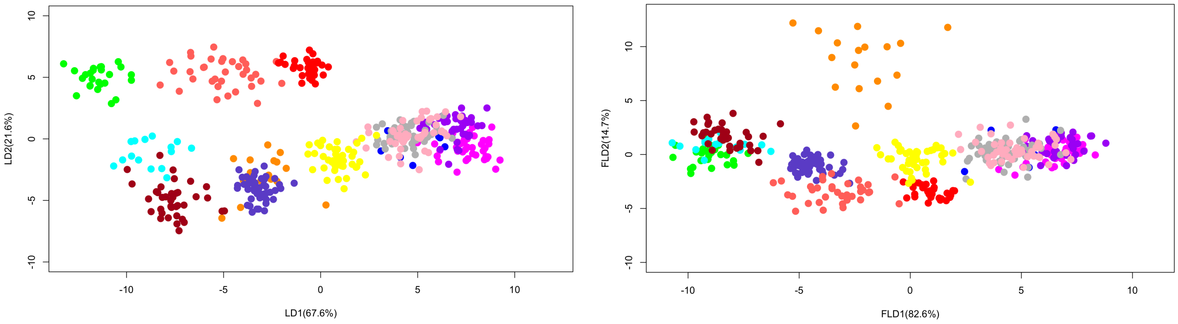

(a) (b)

Supplementary Figure S10. The LDs of the (a) GM (b) FDGM methods.

Results:

- Clusters are better separated using the GM method.
- Proportion of between-class variance explained in the first two linear discriminants is greater using FDGM compared to GM.

Dorsal

PCA

MFPCA

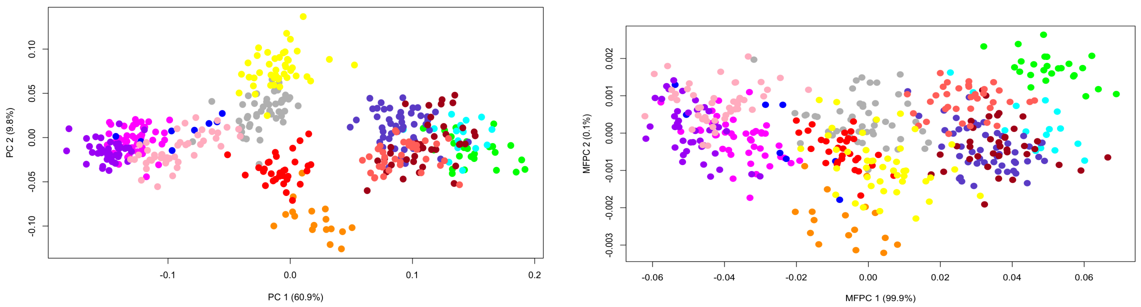

(a) (b)

Supplementary Figure S11. The PCs of the (a) GM (b) FDGM methods.

Results:

- Clusters are better separated using the GM method.
- Variation explained for the first two principal components is the greatest using FDGM.

LDA **FLDA**

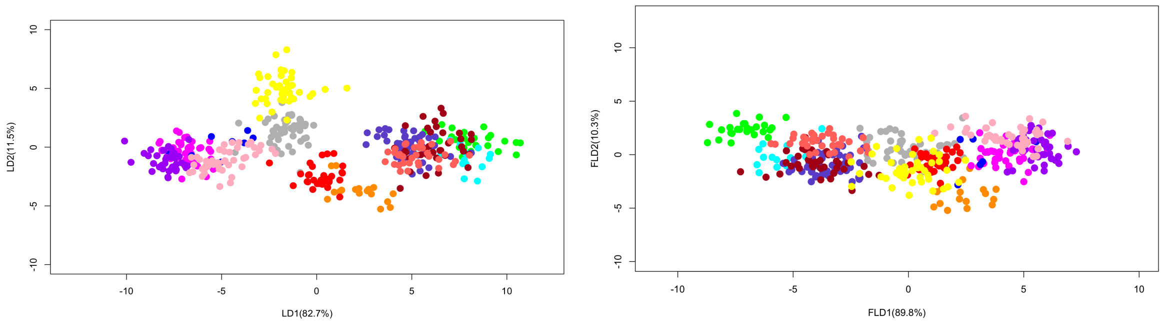

(a) (b)

- bantingensis
- belanaki
- careyensis
- chelatus
- fenestrum
- funnelus
- grandis
- johorensis
- kedahensis
- kederai
- liewi
- navjotsodhii
- parvicopulatrix

Supplementary Figure S12. The LDs of the (a) GM (b) FDGM methods.

Results:

- Clusters are better separated using the GM method.
- Proportion of between-class variance explained in the first two linear discriminants is greater using FDGM compared to GM.

Machine Learning

Supplementary Table S3: The mean accuracy on the test sample and the corresponding standard deviations (in brackets) based on 20 replications using the MFPCA and PCA scores for: (a) dorsal and (b) ventral.

Classifiers	Mean	
	**GM**	**FDGM**
NB	0.816 (0.034)	0.762 (0.048)
SVM	0.855 (0.008)	0.793 (0.007)
RF	0.853 (0.010)	0.763 (0.007)
GLM	0.848 (0.005)	0.811 (0.005)

(a)

Classifiers	Mean	
	**GM**	**FDGM**
NB	0.771 (0.030)	0.767 (0.032)
SVM	0.758 (0.013)	0.794 (0.008)
RF	0.752 (0.009)	0.750 (0.010)
GLM	0.727 (0.013)	0.654 (0.004)

(b)

Results:

- Overall, the results obtained for the FDGM is comparable with GM.
